# The semantic architecture of the World-Wide Molecular Matrix (WWMM)

**DOI:** 10.1186/1758-2946-3-42

**Published:** 2011-10-14

**Authors:** Peter Murray-Rust, Sam E Adams, Jim Downing, Joe A Townsend, Yong Zhang

**Affiliations:** 1 Unilever Centre for Molecular Science Informatics, Department of Chemistry, Lensfield Road, Cambridge CB2 1EW, UK

## Abstract

The World-Wide Molecular Matrix (WWMM) is a ten year project to create a peer-to-peer (P2P) system for the publication and collection of chemical objects, including over 250, 000 molecules. It has now been instantiated in a number of repositories which include data encoded in Chemical Markup Language (CML) and linked by URIs and RDF. The technical specification and implementation is now complete. We discuss the types of architecture required to implement nodes in the WWMM and consider the social issues involved in adoption.

## Origins/history/vision

The World-Wide Molecular Matrix (WWMM) was conceived in 2001 in the spirit of the about-to-be launched UK eScience [1] programme and also the rapid and exciting success of peer-to-peer (P2P) systems in the music industry, such as Napster [2]. We interpreted the spirit of the age to be the dawn of a data- and knowledge-rich infosphere which would be self-evidently valuable to science and where every discipline would be actively publishing their data on the web. The vision was also inspired by the cyberpunk of William Gibson [3] and others with his idea of the information matrix where humans and machines would "jack-in" to an essentially infinitely large amount of information resources. This vision was 20 years ahead of its time but besides coining the term "cyberspace", now has many features of today's evolving web ("semantic web") communities. It is from this, and not from the Matrix films [4], that the word is borrowed with thanks. The concept is sufficiently compelling that others outside this group have set up a Wikipedia article on the WWMM [5]. Inspiration was also provided by the final session at WWW1 [6] (1994) where Tim Berners-Lee outlined brilliantly how semantic information would drive and represent events in the real world, and the WWMM has tried to capture this for the domain of chemistry and related sciences.

We have often used the term "chemical semantic web" which is effectively synonymous with the WWMM, the preferred term in this article.

This article describes the evolution of the WWMM. Some of the early ideas (several of which were exposed in the eScience program) were ahead of implementability but are now linked into general semantic web approaches. The paper therefore represents an evolving vision of a distributed decentralised system.

The eScience programme held out the vision of a total network ("Grid") of linked computing resources, with provision for high-speed access and interchange of data. We assumed that this would be a semantic network where many of the resources would not be bytes and CPU but would be structured information. We were grateful to receive early funding from the eScience project ("Molecular Standards for the Grid"[7]) but have been somewhat frustrated by the top-heavy concentration on CPU performance, bulk storage of un-semantic data and almost obsessive concentration on building middleware. The eScience programme, *per se*, contributed little to the semantic web in our fields.

Like many early ideas, it is impossible to predict the requirements for successful autonomous growth and it has taken approximately 10 years for the initial ideas of the WWMM to become an early reality today. The semantic web and, in its wake, the WWMM, have had to wait for the time to be right for them to flourish. This requires a complex mixture of different requirements:

• A widely-distributed toolchain in at least alpha.

• A critical mass of early adopters.

• A general realisation that this was an imperative whose time was bound to come.

In bioscience these ideas have been taken up at an early stage and many semantic resources have been created. There is a large amount of public investment in bioscience information technology driven in part by the Genome publications but also by the realisation that machines were going to be essential for discovery linking and simple inferences from semi-structured knowledge. We believed, optimistically and perhaps naively, that the same philosophy would be taken up in chemical disciplines. Some chemists had led the field of AI in the early 1970s (DENDRAL and CONGEN [8], LHASA [9]) and it was natural to assume that chemistry would be a growing point for the semantic web.

In fact, there have been relatively few new conceptual developments in mainstream chemical informatics over the last decade or more. Apart from the development of InChI [10] (a semantic identifier system for connection tables), there has been very little central community interest in creating semantic resources. Many businesses and information providers take a 1980s model of capturing data (expensively), packaging it and re-selling it to the community. Similarly almost all publishers of chemistry are closed access and have determinedly remained so. This means that the data deluge expected in 2000 has failed to materialise in chemistry. The consequence of this is that not only is there no data to make semantic, there is little understanding in the community of the value of semantic data.

This situation is now changing. The semantic web is now reaching the high street and powerful commodity tools can be used for managing distributed linked data. Chemistry cannot ignore these developments. The "walled garden"[11] model of data is being shattered in governments, geospatial systems, music, libraries *etc*. where institutions are realising that to fulfil their roles they need to make their data Open and to make it semantic. There are still major cultural social commercial and political barriers; for example, the automated machine extraction of chemistry from electronic articles may result in a legal action by the publisher, and this attitude has held back the development of the WWMM by a considerable period.

In 2000, we envisaged that the technology would be based on P2P systems, where all nodes in the network would be equally able to receive and publish semantic data. The current evolution of the WWMM has been strongly influenced by the technologies in common everyday and business use, and now is much more likely to consist of servers and clients using REST (REpresentational State Transfer)[12] and similar philosophies for information exchange. The original vision however of a community-led process, sharing resources, is still at the heart of the WWMM.

The Napster and similar models worked because of a fortunate combination of circumstances. Almost all nodes were read-and-publish, in that they would consume information they wanted (music tracks) and would install a re-publication server as part of their "bargain" to the community. In addition, the metadata for music is relatively simple and was already widely used. The title of a track or artist generally identifies more or less precisely what is required. The P2P model survives in systems such as Skype [13] and BitTorrent [14] where owners of clients are prepared to pay for benefits in kind through offering bandwidth and services. A necessary requirement is that software is available which is almost transparent for the client to install and re-use.

The WWMM started with a more complex challenge. The metadata for molecules (and even more, chemical reactions, substances and properties) is not as simple as discovering music on the web. But the biggest challenge was that software would have to be written, which could be trivially distributed and where clients could legitimately and safely offer services without needing to know the details of installation. Nevertheless, the original (2001) concept has lost none of its validity. We envisage an ecology of sites (using a common syntactic and semantic infrastructure) which store a variety of objects in different numbers and with different attributes, and offer them to the world for re-use. Some sites can be expected to provide monocultural collections of certain types of object (*e.g*. molecules) while others might represent the work created in a particular institution. We also expect that there will be specialist sites for aggregating and indexing. This is a potential model for publication of data and metadata. In 2004 we had anticipated that some of the roles of the WWMM would be exemplified by the infrastructure and ecology of university institutional repositories but in reality these are poorly linked and there is no re-use and re-purposing of content.

It has become clear that in science domain-specific repositories are the appropriate model and in several fields there is a critical mass of adoption, support and contribution of content. Many of the bioscience repositories are managed by international data centres such as the NCBI (National Center for Biotechnology Information)[15] and EBI (European Bioinformatics Institute)[16], but a newer generation of distributed, often university-based domain repositories are emerging. Two examples of these are Dryad [17] (where ecological content is deposited) and Tranche [18] (where proteomics data such as mass spectra are deposited [19]). These models are particularly compelling as it is now a requirement of several journals and publishers that data is committed to them. The WWMM is a technology that can respond to such requirements in chemical publishing.

By contrast, in chemistry, the only mandatory deposition of domain-specific data is in crystallography. Some of this is published openly on publishers' websites (and we use this in CrystalEye [20]), but approximately half of it is deposited directly in the CCDC. This has been a pioneering example of a domain repository but is now hampered by the fact that the data are not Open. While individual crystal structures can be requested by email, a considerable proportion of the raw data (in major journals) are only accessible in bulk by subscription. There are also restrictions on the re-use and re-publication of this data.

In science, repositories seem to work best where there is a central unifying concept found in every entry. For example, Swiss-Prot [21] is based on protein sequences, PDB [22] on protein structures and GenBank [23] on nucleic acid sequences. This may be, in part, because the repositories represent well-accepted concepts in the discipline and in most cases have an organisation or a committed group who oversees the semantics and ontology. It is necessarily a reductionist view and considerable flexibility and detail is lost, but at this stage in scientific information it is vastly better than having nothing at all.

## Semantics and Ontologies in Molecular Sciences

The major current repositories of chemical information are generally run outside the community input of chemists and related disciplines Very few are fully Open (exceptions being bioscience-based collections of molecules *e.g*. PubChem [24], ChEBI [25], NMRShiftDB [26] and CrystalEye, and the emerging collection in Wikipedia). There is a limited amount of Open data in ChemSpider [27] but Chemical Abstracts asserts copyright over its identifier system, does not publish its ontologies, and charges for lookup of names and identifiers.

For a full semantic implementation we need a variety of identifier systems with ontological mapping between them. We expect that, at some time, chemistry will develop a semantic infrastructure similar to that in current bioscience. In 2011, we note the development of semantic resources between Southampton and ChemSpider, but in general there is conservatism and resistance to the free flow of chemical information, and hence to the development of infrastructure. We have therefore taken a pragmatic view that much of what we implement can be done without formal ontologies and supported by a dictionary concept (see article in this issue).

Identifier systems are essential but very challenging. Semantic identifiers will always fail to represent general concepts, because the decision of which aspects of the concept are important to its identity are fixed, or from a fixed set (*e.g*. InChI). InChI doesn't fall short because of which information it chooses to include, it falls short because it chooses. In contrast, the CAS system is more flexible and can be assigned to a wide range of chemical substances. We recommend the use of relatively short alphanumeric identifiers. Chemists have a long tradition of using numeric identifiers (*e.g*. CAS, in-house compounds, regulatory labels *etc*.) and for most systems sequential numbering seems to be the best way of minting identifiers.

Arbitrary identifiers, however, require a central authority (even if only a server to mint the next in sequence). Without this, name collisions are certain. Moreover, without authorities to maintain identifier systems, they inevitably decay. We hope that this paper may stimulate persistent non-profit organisations (such as international scientific unions and learned societies) to create Open identifier systems. In the absence of this, the most likely solution will be through web persistence as in Wikipedia (though we note that that does not yet have a unique identifier system, being based completely on linguistic approaches).

In chemistry the "molecule" has become a central concept for aggregation. We note that there is much semantic and ontological confusion between substance, compound, connection table, and other concepts describing chemical objects and their composition. Thus, for example, the InChI only formally relates to a connection table, and works where there is a pragmatic correlation between connections tables and the composition of substances. It breaks down where a substance may contain components with different connection tables, where the connection table is dynamic, or where different substances can occur in different macroscopic forms. The technology of WWMM can support concepts such as molecule (connection table) and substance independently.

The WWMM paradigm relies on a unique identifier system for discovering and asserting the identity of objects. This works well where the connection table is a complete description and identification of the substance, but where it fails (*e.g*. "aluminium chloride", "glucose", "diamond") we must rely on an authority to provide a controlled identifier system. The system in commonest use is the Chemical Abstracts registry number (CAS number)[28] but this is not Open and its use outside CAS is restricted to a small percentage of the compounds indexed by CAS. The best candidate for an Open system of substance identifiers is Wikipedia, which at the moment uses textual representations as the public unique identification of pages describing compounds. Until there is a public identifier system, the WWMM concept will be restricted to entries where connection tables suffice.

**Figure 1:**
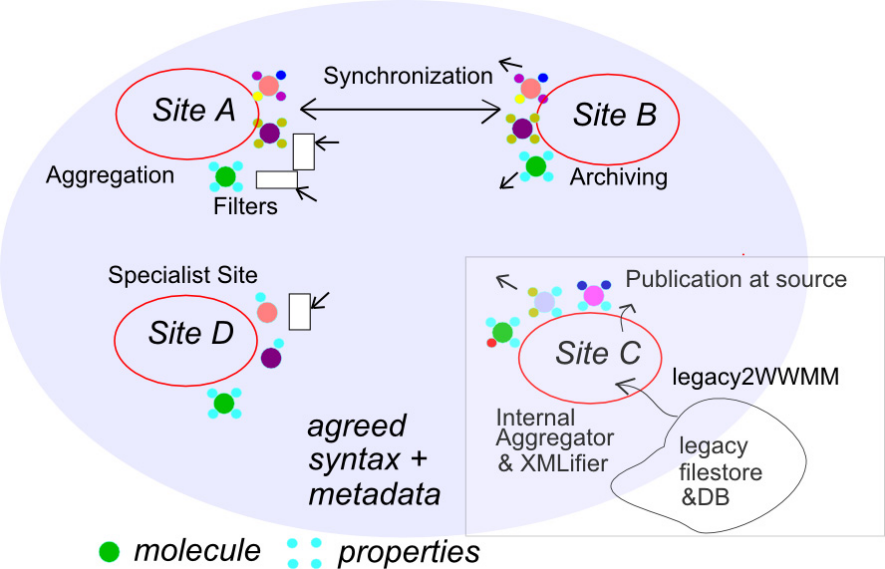
**A 2004 vision of the variety of functions in the distributed sites of the WWMM concept**.

Figure 1 shows four sites all playing different roles in the WWMM. Site A is an aggregation site which trawls the web, either for other WWMM sites or legacy (white rectangles) and aggregates this in a similar manner to conventional search engines. The objects aggregated in the diagram are molecules with a variable number of properties (physical chemical and metadata). The concept can be extended to other chemical objects such as crystals, spectra, reactions and computational chemistry. In some cases we would have single instances of an object with several different properties (site A, right), while in other cases an object would be observed several times and have different instances of the properties (site A, bottom). Site B represents an archival site (*e.g*. the Internet Archive's Wayback Machine [29]) where it would mirror for posterity the transient picture on site A. Site C represents data publication at source (*e.g*. our current CLaRION project [30] which is designed to publish scientific data from the laboratory to the web). The expectation is that visitors to the site (machines or humans) can then either assess the value of the site itself *e.g*. for data-oriented peer review, or can aggregate and re-use objects of interest. Site D specialises in one particular facet of objects or properties. This is exemplified by CrystalEye which trawls the web and extracts only crystal structures and collates and systematizes them.

This vision, in 2004, was ahead of the technology to implement it, although we created some early prototypes of parts of the system. In both closed and Open systems, the successes have largely been through centralised sites (*e.g*. Google, Open StreetMap [31], ChemSpider, DBpedia [32]). These have the value of coherency and visibility but can run into problems of scale and also potential frustration with central control. The P2P system is more flexible and allows a different type of innovation but is harder to reach to critical mass. It represents a general imperative for the web, a distributed non-hierarchical system of sites collecting and publishing data. This architecture is reflected in both the Quixote project [33] and the OpenBibliography project [34] reported elsewhere in this issue. It is clearly difficult to create off-the-shelf software for these types of system, but we believe that an investment in RDF, a very strong investment in all types of metadata in the system, and, most importantly, a critical mass of a community prepared to explore this will come up with prototypes which show the value.

The WWMM is also designed to hold properties of chemical molecules and substances. In many cases, these concepts are very well defined and managed by community definitions such as the IUPAC Gold Book [35]. However, there is much opportunity for confusion: scientific units of measurement are often omitted and physical constraints (*e.g*. pressure at which a boiling point was measured) are not recorded. In some cases it is unclear what the molar unit is. For example, some programs calculate the extensive properties for a complete unit cell (*e.g*. Na_4_Cl_4_). These properties are supported by a system of dictionaries (see the sibling article in this issue).

Concepts which are relations between objects (*e.g*. chemical reactions and processes, such as chemical syntheses) have been excluded from the initial version of the WWMM until their semantic representation has been more explored within the community.

In a distributed system, there is a major challenge of different versions of the "same" object. Traditionally and currently many systems tackle this by creating a canonical "correct" object by merging different versions into one. Systems such as CrystalEye work well because although there are a variety of sources, there is only one agreed instance of the crystal structure publication. Sites such as ChemSpider normalise chemical names and identities by correcting "wrong" names and structures. Building a more complex system than this is psychologically difficult with the dangers of either over-simplistic representation through normalisation or over-complication of the details of different occurrences of objects. A typical problem is the management of the different versions over time and location of human-authored documents.

**Figure 2:**
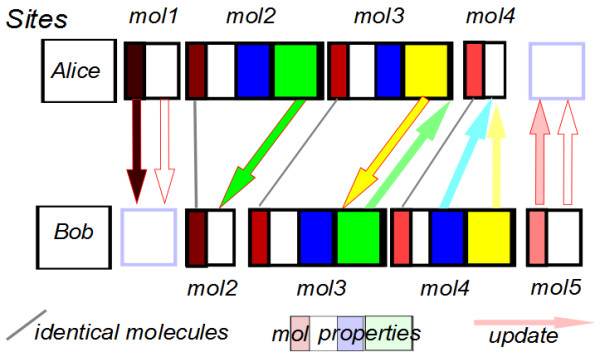
**An example of the problems of different entries and different versions in two repositories**.

Figure 2 illustrates this problem: mol1 exists in Alice but not Bob, and mol5 exists in Bob not Alice. mol2 exists in both, but Alice has more properties (attributes), and mol4 is the reverse. mol3 is identical in both repositories. The arrows show various updating processes so that Bob will need to import mol1 and all its properties to be in sync, and Alice must do this for mol5. For mol2 and mol4 each site would have to import properties and keep them in sync, whilst for mol3 only the values of properties need to be synchronised. In practice it is likely that Alice and Bob will not synchronise at this level and it is up to users of their sites to determine existence of entries and of properties, and the identity relations.

Because of this, we think that the CrystalEye and Quixote systems are excellent example of systems that can succeed as distributed WWMM repositories. In CrystalEye the uniqueness is determined by the bibliographic data of the publication (or the metadata from the creators). In Quixote a calculation is the same regardless of which laboratory carries it out and duplications of calculations have the same canonical representation. We believe that there will be a demand for molecules from CrystalEye and Quixote and that these will be excellent exemplars and workbench for crystallographers, scientists and computational scientists interested in P2P systems and distributed repositories.

The Linked Open Data (LOD) concept and movement has demonstrated the vision of a cloud of interlinked resources, and many of the bioscience databases feature prominently. In 2011, there are still very few Open data resources in chemistry. To be a full member of this graph, a resource has to have a public identifier system (URI) and a license that allows essentially total freedom of access and re-use. "Free resources" (where there is no right of re-use) cannot be included. The following current resources could be transformed into LOD nodes:

• Bioscience databases (PDB, Uniprot [36], KEGG [37]*etc*.)

• NMRShiftDB (a volunteer-driven collection of Open NMR spectra)

• A subset of the ChemSpider resource (a small percentage of items are now labelled as 'Open Data' and there is a stable identifier system)

• Chemical entries in Wikipedia

• CrystalEye (semantic crystal structures from Openly published data)

• Computational chemistry from the Quixote project

The data so far has been primarily from current aggregators and voluntary collections. The WWMM concept also included the idea that scientists would publish their data directly onto the web as they carried out experiments or calculations. Although a very small percentage of the community, chemistry has been among the leaders in developing this idea and J-C Bradley [38] and, more recently, Matt Todd in Sydney [39] and the Frey group at Southampton [40] have published tools and data onto the public web. In particular, Todd's community of collaborative drug design has attracted considerable interest and, assuming it is successful, will be a strong driver to show the value of the semantic web and WWMM approach. The concept of "the contents" of a site can be problematic. At one level, chemists think of collections of molecules as a large defined collection of molecular datafiles which could in principle be distributed on a memory device or published as individual pages on the web. In other circumstances, molecules and their properties are retrieved from a search system. In yet other applications, molecules can be generated "on-the-fly" by web services (an example is our OPSIN server [41] which converts IUPAC names into connection tables and effectively has an infinite number of possible molecules). In many closed systems it is impossible to tell whether particular data is in the system unless the interface allows us to ask this question. The WWMM is conceptually designed as an infrastructure where all of the content can be systematically retrieved and the limitations are technical rather than socio-political.

## Design and evolution: technologies

Tim Berners-Lee originally introduced the four principles of linked data [42]:

1. Use URIs as names for things.

2. Use HTTP URIs so that people can look up those names.

3. When someone looks up a URI, provide useful information, using the standards (RDF*, SPARQL).

4. Include links to other URIs so that they can discover more things.

The modern WWMM adopts principle 1 completely. All things including not only data but metadata such as dictionaries are completely supported by URIs. Principle 2 brings certain problems. In the initial design of HTML and XML there was a strong architectural differences between URLs (addresses) and URIs (identifiers) and this formal distinction has to remain in many fields. Tim Berners-Lee simplified this to principle 2 on the basis that everything of interest could have both an address and a URI, and that they could be conflated into the same string. For this to happen, the identified object must be sufficiently stable and conceptually bounded that it is effectively describable as a single persistent object. (There are ontological systems which can describe non-persistent and mutable objects but they are beyond the current scope of chemistry and the WWMM.) The single address requirement can also be problematic. Principle 2 only fails to break when the user or user agent has pervasive access to the web (*e.g*. not in an aeroplane) and where the maintainer of the resource can guarantee 24/7 availability. If this latter condition cannot be met, then either the system breaks (perhaps temporarily) or it has to provide a fall-through mechanism of aliased addresses. CML was originally designed with the clear W3C principle that names and addresses were distinct but we are attracted by the conflated URI vision which we believe will work for much of chemistry. Given at least a partial implementation of principle 2, then we endeavour to satisfy principle 3 by using RDF and SPARQL where appropriate. Principle 4 is a fundamental part of the WWMM and follows practice in, for example, bioscience where most resources have copious links to others. Whether or not resources are normalised is a problem that we have not yet explored in depth.

More recently, discussions on the eGov W3C mailing list [43] refer to Tim Berners-Lee's "five star" model for government data:

* on the web, open license

** machine-readable data

*** non-proprietary formats

**** RDF standards

***** Linked RDF

Semantic data requires a minimum of an identifier system. Many published collections of information do not generate identifiers and are only accessible and identifiable through their web addresses. This is a fragile design and it is essential that components of the WWMM have unique permanent identifiers. The traditional use of chemical identifiers has been restricted to large authorities such as CAS, Beilstein [44], RTECS [45], and more recently, ChemSpider, PubChem, DrugBank [46], ChEBI and ChEMBL [47]. Of these, we believe that only the bioscience-oriented systems (ChEBI, ChEMBL, PubChem) are formally Open (*i.e*. that the whole identifier system, with or without the data, can be re-used without permission). There are a small number of spectral identifiers in NMRShiftDB and a small number of reaction identifiers in KEGG, confined to biological transformations. The CrystalEye collection does not have an identifier system yet although the Crystallography Open Database (COD)[48] does. There is no Open system for small molecule crystallographic identifiers (the CCDC [49] codes are for a closed system).

In principle, LOD can be completely held as RDF triples. However, many components of chemistry (molecules, spectra, reactions *etc*.) are more easily understood and processed in XML form (*e.g*. CML). The WWMM, therefore, is a mixture of CML components linked together and annotated by RDF triples. As the semantic web develops new approaches to indexing and describing RDF we can expect the flavour of RDF to evolve. In 2011, it is still unclear exactly what triple-store or other RDF technology is required to support large amounts of RDF, but we believe that for local collections (*e.g*. the output of a laboratory) there are now many good OS RDF engines.

We have built prototype ontologies with formal RDF-based systems such as OWL [50], and developed an OWL-based system (ChemAxiom [51]) which describes physical properties and aspects of chemical structure and composition. At present, however, we believe the implementation cost (validating the ontology, installing sufficiently powerful servers) not to be cost-effective. This parallels our experience in Open Bibliography (see sibling article in this issue) where the implementation costs were too large to be deployable without additional resource, and we reverted to a simpler model with some implicit semantics. There is also a psychological barrier in that many scientists working with chemical information need to feel comfortable with the textual representation.

To be semantic, the information must be understandable by machines and humans. In the full semantic web vision, this is (partially) provided by high-level ontological frameworks such as OWL, OBO [52], Cyc [53]*etc*. In WWMM we take the view that semantics can be provided by a number of inter-operating dictionaries which describe the semantics in human terms and also provide a variety of machine-enforceable constraints and interpretations. These work at a pragmatic rather than a formal level. The success of the WWMM will depend in part on the willingness of the community to create such dictionaries and to make sure that material produced uses the dictionary URIs in its annotation. Unlike all current knowledge bases in chemistry, the WWMM will not have a central repository and service. Like peer-to-peer systems we expect that there will be a federation of repositories adopting common identifier systems and semantics. We do not believe that traditional institutional repositories are the most appropriate place to deposit scientific data, and strongly believe that domain-oriented approaches are required. A scientist wishes to interact with a repository that understands her problem, not with the organisation that happens to employ her. Because chemistry is a multi-disciplinary subject we expect that the WWMM will consist of a considerable number of independent nodes. There is no requirement that any given repository holds "all" the data, nor that data should not be duplicated in different nodes. We expect that the community will evolve systems that make sense in terms of ease of access and robustness.

It will be fundamental to have an indexing and discovery system. Because of the non-textual nature of much chemistry, current search engines such as Bing and Google will not be able to index much of the WWMM material. We therefore need distributed search technologies and in the first instance will rely on RDF and on conventional chemical substructure search. We have designed the system such that it is possible for scientists to add indexers (plug-ins) to a repository to create domain-specific searchable metadata. For example, it is not easy to search on the web for a compound containing between 10-15 carbon atoms, but if a repository exposes a carbon-count field as RDF then it is straightforward to retrieve entries using an RDF query containing combinations of index fields. More complex chemical concepts can also be indexed, such as peaks in NMR spectra, cavities in crystals or HOMO-LUMO gaps in theoretical calculations.

The architecture of the WWMM is built on a number of web standards and protocols, described in detail below:

**• SWORD deposit**: publish data to server

**• Atom archive feeds**: syndicate published data

**• HTTP content negotiation**: retrieve data in human and machine understandable formats

**• OAI-ORE/RDF: **machine understandable representation of the data

### SWORD/AtomPub

The Atom Publishing Protocol (AtomPub [54]) provides a standardised application-level protocol for publishing and editing Web Resources using HTTP. AtomPub is applicable to many domains, but is particularly widely supported by the Blogosphere, where it enables authoring tools such as Microsoft Word to publish content to different blogging software using a common protocol. The JISC-funded SWORD (Simple Web-service Offering Repository Deposit) project [55] extends the AtomPub protocol to support the deposit of aggregate resources - packages consisting of a number of related files and associated metadata - onto a server. For example, the 'package' object needed by WWMM may include crystal structure (CIF and CML formats), picture of the 3D structure, and a 2D representation of the connection table *etc*.

### Atom/RSS Feeds

**Figure 3:**
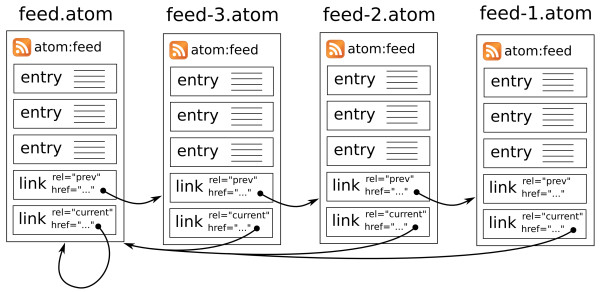
**An example of "paging" using Atom feeds**.

**Figure 4:**
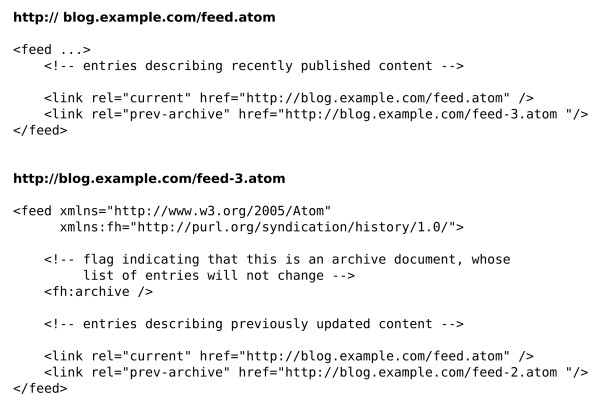
**Atom feed content, based on the example in Figure 3 above**.

Web feeds are widely used to provide users with notifications of updated content. Typically a feed document lists recent content - such as active news items, or the list of articles in the current issue of a journal - and by monitoring ("subscribing to") a feed, users can be alerted when new content is published. Entries in a feed document typically contain the title and summary of an item, along with a link to the full resource. Earlier iterations of the WWMM made use of RSS feeds [56] to alert users of newly published chemical data, but these suffer from the constraint that only the most recent content can be accessed. The Atom Syndication Format offers a solution to this limitation through standardized support for paging, specified by RFC5005. Like an RSS feed, the Atom feed's document contains a list of recently updated content, however it can also contain a link to a previous page containing entries describing other content. A client application can always access the latest content by retrieving the document at the feed URL, but can 'walk' back through the previous pages to discover all the content in the system (Figures 3 and 4).

### HTTP Content Negotiation

**Figure 5:**
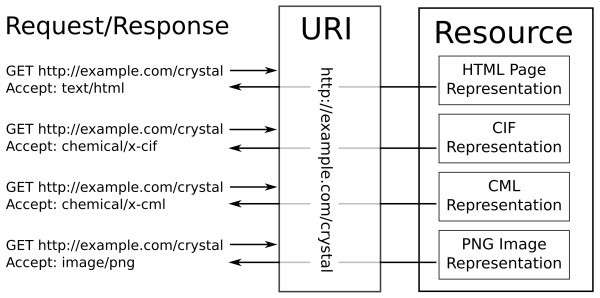
**HTTP content negotiation delivering different representations of the same URI, based on the content of the 'Accept' header**.

The HTTP protocol [57] allows content providers to deliver alternative representations (*e.g*. multiple languages, data formats, size, resolution *etc*.) of a resource (*i.e*. a data object or service identified by a URI) from the same URI, based on the preferences expressed by a client, through a mechanism called content negotiation (Figure 5).

When requesting a resource from a web server, a client may include an 'Accept' header in the request, indicating the media types it prefers, and optionally a strength of preference; for example, Mozilla Firefox 4.0.1 uses the following header:

Accept: text/html, application/xhtml+xml, application/xml;q = 0.9, */*;q = 0.8

This says that Firefox prefers HTML (text/html) and XHTML (application/xhtml+xml) content, or less strongly (q = 0.9) XML (application/xml). If none of these are available it will accept anything else (*/*).

The WWMM uses content negotiation to publish data in formats that are both human and machine readable. The URI for a resource published on the WWMM can be resolve to alternative representations - an HTML or XHTML 'splash' page for humans, or an RDF representation (application/rdf+xml) for machines.

This request by a web browser (such as Mozilla Firefox)

GET/crystals/211721 HTTP/1.1

Host: http://crystaleye.ch.cam.ac.uk

Accept: text/html, application/xhtml+xml, application/xml;q = 0.9, */*;q = 0.8

will return a human-friendly HTML page describing the crystal structure, while the following request for the same resource by a machine agent

GET/crystals/211721 HTTP/1.1

Host: http://crystaleye.ch.cam.ac.uk

Accept: application/rdf+xml

can return a machine understandable RDF representation of the data.

### OAI-ORE/RDF

**Figure 6:**
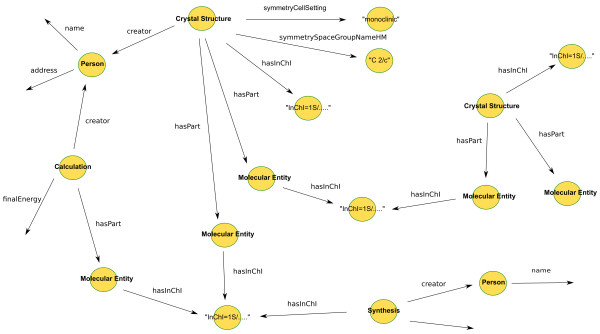
**Using an RDF representation for data items such as crystal structures and calculations enables them to be connected by shared concepts (InChI, creator) to form a graph of linked data**.

Open Archives Initiative Object Reuse and Exchange (OAI-ORE)[58] is a standard for describing aggregations of Web resources, commonly serialized into RDF. The WWMM uses OAI-ORE to describe the resources making up a data item - *e.g*. a crystal structure of NMR spectrum and the aggregate resource (Figure 6).

The OAI-ORE model includes three classes of object: Aggregation, Aggregated Resource and Resource Map. Aggregations are an abstract concept, containing one or more Aggregated Resource. An Aggregation may be serialized into a number of different formats, and each of these serializations is termed a Resource Map. Each Resource Map has a unique URI, distinct from the Aggregation's URI, in order for the different representations of the Aggregation to be resolvable.

**Figure 7:**
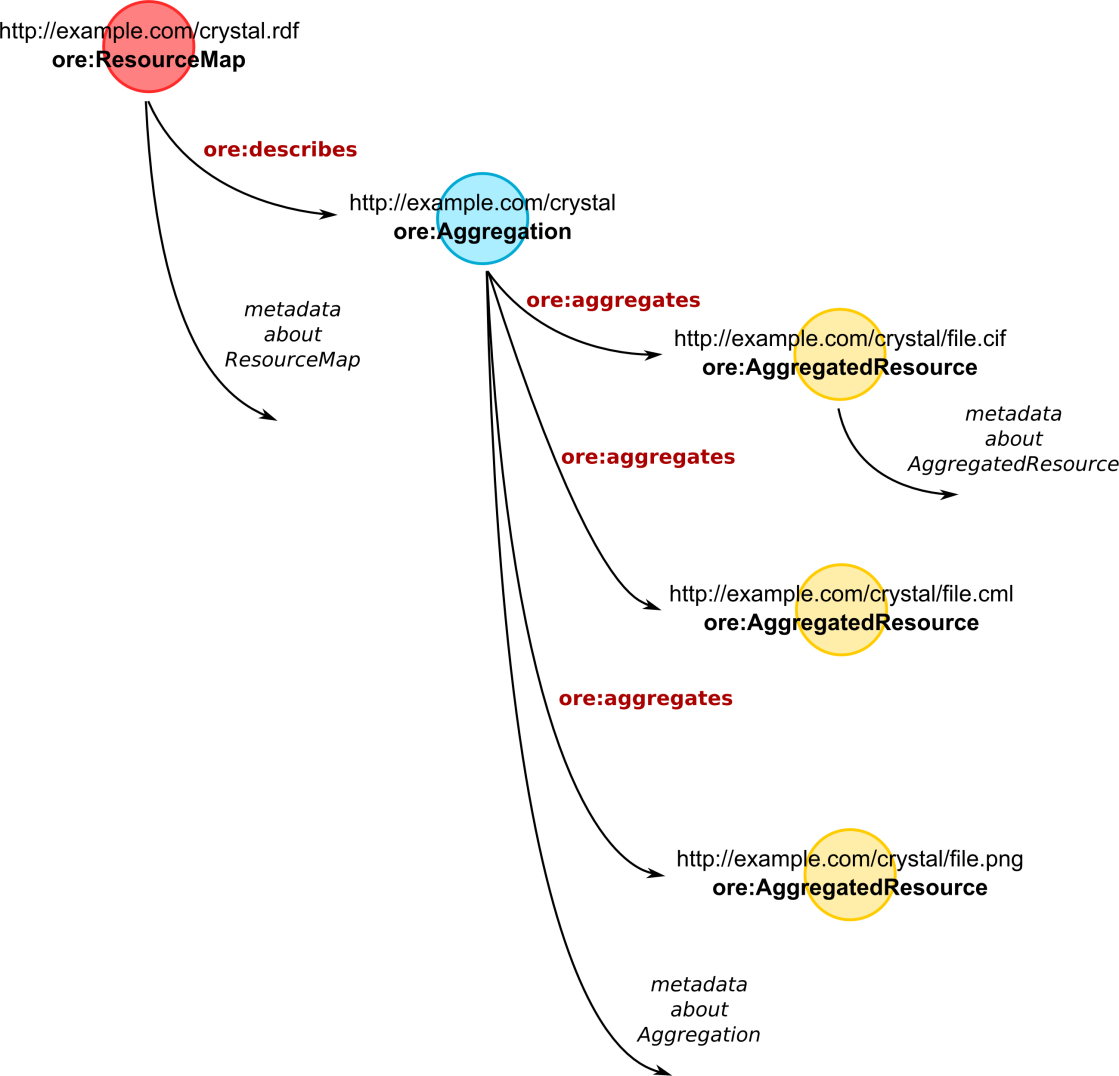
**The structure of an RDF representation of an ORE resource map describing an aggregation of related resources and associated metadata**.

As well as describing an Aggregation, a Resource Map may contain additional data about the Aggregation and the individual Aggregated Resources (Figure 7).

## Software development environment

We have developed a large number of software components of varying complexity with much inter-dependence between the components. We embrace agile development practices and our software development environment is built upon existing technologies. Substantial use is made of existing Open Source utilities, tools and libraries such as Apache Commons [59], Restlet [60] and CDK [61]. The majority of the code is written in Java and we use the Apache maven [62] build system (compiles, manages dependencies *etc*.)

**Figure 8:**
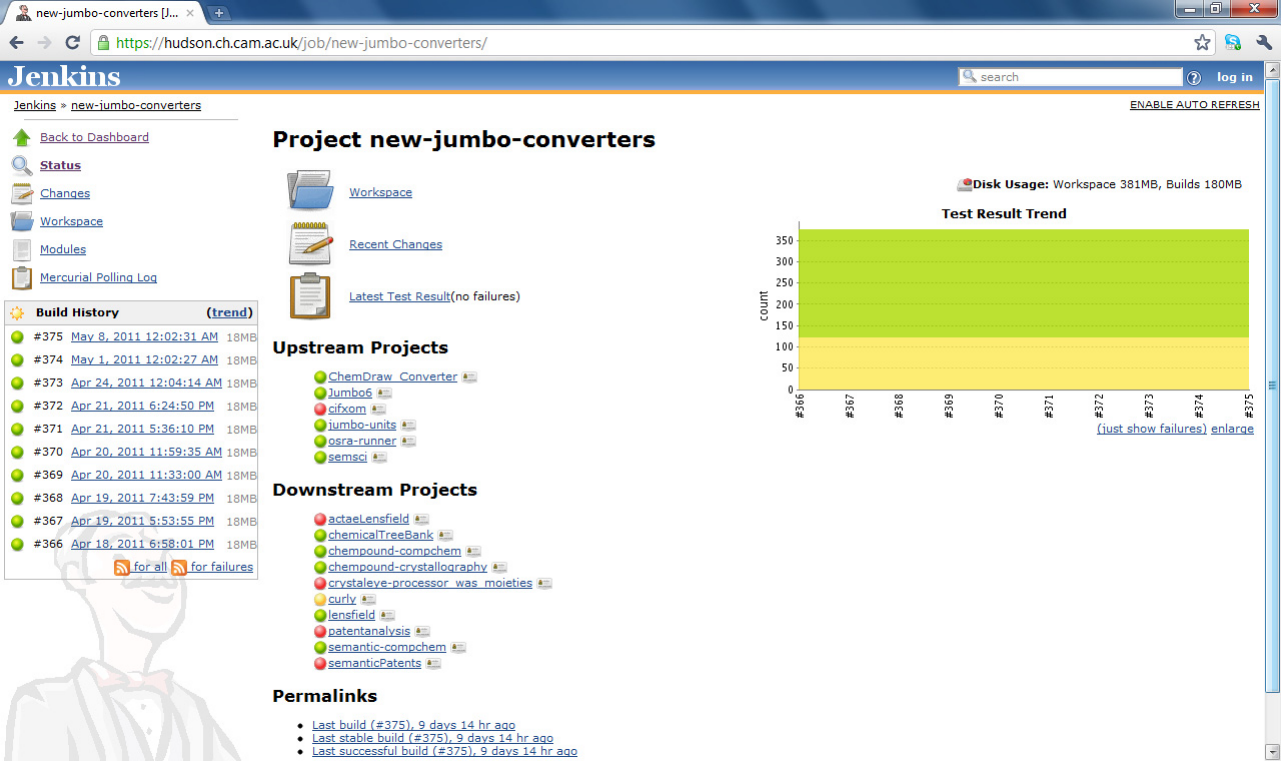
**Current status page of the 'new-jumbo-converters' project on the Jenkins continuous integration server, showing its relationships to other projects**.

We endeavour to write the code with high test coverage (as much unit testing as possible is built-in at the initial stages), and aim for test-driven development. We run a Jenkins continuous integration [63] server and a Nexus maven repository so all the code is developed under source control (a mixture of Subversion (svn)[64] and mercurial [65]). The Jenkins server polls the source repositories at regular intervals and rebuilds and tests any updated projects in a clean environment. If the updated code compiles successfully and passes all the unit tests, it is deployed to the maven repository and any downstream (dependent) projects are then re-compiled/re-tested in the same way. Thus, any modifications which would break compatibility with any other components are flagged, identified and rectified at the earliest possible opportunity (Figure 8).

## Virtual communities

The WWMM is predicated on a critical mass of users who are prepared to develop both content and technology. The precise path for the evolution will depend on a mixture of what technologies are available, the familiarity of the community and the resources that are available. It may also depend on the perceived business models and the uptake by significant producers and consumers. The earliest experiment is in our Quixote community where we are producing semantic computational chemistry and disseminating this from RDF-aware servers. It is likely that different members of the community will play different roles. Some may wish to upload their results to a semantifier which deposits them in a given repository ("push"). Others may wish to aggregate legacy data and re-disseminate it ("pull"). For example, a University Department or group might wish to expose its results on its own webpages to enhance the reputation and provide re-usable material. A national lab might act as an aggregator for a sub-community of scientists (*e.g*. in materials properties prediction).

## Future Development of the WWMM

The future of the WWMM will depend on a number of factors which we cannot predict:

a) The change from "walled garden" providers to Open collections.

b) Citizen science. The most dramatic example in science has been the large collaboration involved in GalaxyZoo [66], and now spreading to other types of activity (Zooniverse [67]). We expect and hope that this philosophy will spread to chemistry and disciplines which require chemistry.

c) The need to link data. Almost all chemical systems at the moment are unsuitable for LOD in both the lack of semantics and the problems of licences. The ChemSpider system is a hybrid in that some of the data are Open and some of the material is exposed in RDF.

d) The realisation that chemistry needs community ontologies.

e) The high and unsustainable cost of closed data collections.

f) The growing dissatisfaction of the upcoming generation of scientists with closed systems.

g) The frustration of the non-academic community in the difficulty of obtaining material published in STM publications.

h) The desire of scientific publishers and editorial boards to publish the semantic data associated with articles.

We understand that the initial collection of 175, 000 molecules in DSpace@Cambridge [68] is regularly used and accounts for ca. 10% of the repository traffic. This further encourages us to believe that decentralised resources are valuable and can be discovered and used by current web technology.

The WWMM is now technically deployable and its critical mass will depend on adopters who need an Open distributed system, and who are prepared to contribute to the infrastructure design, the ontology design and its implementation.

## Competing interests

The authors declare that they have no competing interests.

## Authors' contributions

PMR had the original vision for the WWMM, has participated in and overseen its development and has written the manuscript. SEA has been responsible for much of the current infrastructure and has written the manuscript. OJD has contributed to the development of the infrastructure, provided valuable advice on technical matters and has written the manuscript. JAT was involved in development of the original infrastructure and content production and has written the manuscript. YZ was involved in the development of the original infrastructure and content production. All authors have seen and approved the final version.
